# GeneHunt for rapid domain-specific annotation of glycoside hydrolases

**DOI:** 10.1038/s41598-019-46290-w

**Published:** 2019-07-12

**Authors:** S. N. Nguyen, A. Flores, D. Talamantes, F. Dar, A. Valdez, J. Schwans, R. Berlemont

**Affiliations:** 1 Department of Biological Sciences, California State University Long Beach, Long Beach, California, USA; 2 Department of Chemistry and Biochemistry, California State University Long Beach, Long Beach, California, USA

**Keywords:** Polysaccharides, Metagenomics, Bioinformatics

## Abstract

The identification of glycoside hydrolases (GHs) for efficient polysaccharide deconstruction is essential for the development of biofuels. Here, we investigate the potential of sequential HMM-profile identification for the rapid and precise identification of the multi-domain architecture of GHs from various datasets. First, as a validation, we successfully reannotated >98% of the biochemically characterized enzymes listed on the CAZy database. Next, we analyzed the 43 million non-redundant sequences from the M5nr data and identified 322,068 unique GHs. Finally, we searched 129 assembled metagenomes retrieved from MG-RAST for environmental GHs and identified 160,790 additional enzymes. Although most identified sequences corresponded to single domain enzymes, many contained several domains, including known accessory domains and some domains never identified in association with GH. Several sequences displayed multiple catalytic domains and few of these potential multi-activity proteins combined potentially synergistic domains. Finally, we produced and confirmed the biochemical activities of a GH5-GH10 cellulase-xylanase and a GH11-CE4 xylanase-esterase. Globally, this “gene to enzyme pipeline” provides a rationale for mining large datasets in order to identify new catalysts combining unique properties for the efficient deconstruction of polysaccharides.

## Introduction

Glycoside Hydrolases (GHs) are Carbohydrate-Active Enzymes (CAZy) that catalyze the hydrolysis of the glycosidic linkage in polysaccharides (e.g., cellulose, chitin) and oligosaccharides (e.g., cellobiose, chitobiose)^1^. GHs are found as single domain proteins (SDGHs) or associated with accessory domains such as carbohydrate binding modules (CBMs) within multi-domain GHs (MDGHs)^2^. In MDGHs, CBMs enhance enzyme-substrate interaction by anchoring the catalytic domain to the substrate^3^. The anchoring reduces diffusion from the substrate and locally increases the concentration of catalytic domains^4^, thus improving the overall polysaccharide degradation^3^.

GHs support essential processes for ecosystem function and for biotechnology. Among others, in land ecosystems, the deconstruction of plant biomass by microbial GHs is essential^5,6^, whereas the breakdown of chitin, from arthropods and fungi, is important in both marine^7,8^ and terrestrial ecosystems^9–12^. Next, in the gut of animals, microbial GHs target polysaccharides, supplement the lack of endogenous enzymes^13,14^ and thus contribute to the processing of complex carbohydrates during digestion^15–18^. Finally, GHs are essential for the biofuel industry, as plant based polysaccharides constitute a major source of sustainable and renewable material capable of providing liquid transportation fuel^19–22^.

Many GH-genes and proteins have been identified in a growing number of sequenced genomes and environmental samples thanks to the use of activity-driven screening^23,24^ and bioinformatic annotation systems^12,16,18,25^. The precise identification of GH-genes and proteins is essential in order to understand how microbes support key functions across ecosystems^17,25,26^ and to identify new enzymes for biotechnological application^18,21,27^.

In order to identify new catalysts for biomass degradation, we examined the performance of sequential Hidden Markov Model (HMM) identifications^28^ combined with publicly accessible HMM-profiles from the PFam database^29^, here referred to as the GeneHunt approach^2,30^, to detect GH-sequences and investigate their detailed architecture (i.e., the precise domain organization of MDGHs)^2^. More precisely, we first validated the GeneHunt approach by re-annotating the biochemically characterized GHs listed on the CAZy database (as of June 2018)^1^. As described for cellulases, xylanases, and chitinases^30^, we expected the PFam-based annotation to correctly identify most of the proteins from the major GH families, although rare and recently identified GH-families would display inconsistencies. Next, we identified GHs in the M5nr database (version 13.12.15) containing 43,098,145 non-redundant, mostly microbial, protein sequences^31^. This collection of sequences derived from the major sequence database serves as the reference database for the MG-RAST annotation pipeline^31,32^. Finally, we identified the detailed multi-domain architecture of GH proteins in assembled, publicly accessible, metagenomes from MG-RAST. We hypothesized that, across database, GH proteins would exist primarily as single domain enzymes with low frequency of MDGHs as identified in sequenced microbial genomes^2,10^. We also expected that identified MDGHs would mostly consist in association between GH domains and CBMs as identified in the CAZy database^1^. Among the MDGHs, we expected to identify proteins with multiple catalytic domains and identified as potential multi-activity GHs (MAGHs). These MAGHs would display multiple catalytic domains with potential synergistic activities. In these proteins, synergistic interactions between catalytic domains would result from the complementarity of the associated domains and from a proximity effect^3^. More precisely, we envisioned (i) parallel pathway synergy where the combined catalytic domains target distinct, yet physically associated, substrates (e.g., cellulase:xylanase) and (ii) debranching synergy where one catalytic domain cleaves the side groups in substituted polysaccharides or cleaves branch points in reticulated polysaccharides, thus increasing the accessibility of the polysaccharide backbone for the second catalytic domain (e.g., xylanase:xylan-esterase). Relative to SDGH and MDGH (with CBMs), these MAGHs represent vastly untapped enzymatic diversity with great potential for improved biomass deconstruction^21,33–35^.

Globally, this work provides the rationale and validation for the detailed and rapid detection of most identified GH families and associated domains from a variety of datasets, ranging from biochemically-characterized enzymes, the largest non-redundant sequence database, and assembled metagenomes derived from various environments. In addition, this work provides an exhaustive list of domains associated with GH domains, explores the diversity of multi-domain and multi-activity GHs, and identifies new types of catalysts with potential for biotechnological application.

## Results

### Mapping of glycoside hydrolases in CAZy and M5nr

First, in order to evaluate the GeneHunt approach, we (re)annotated the sequences of biochemically characterized GHs listed on the CAZy database. GeneHunt consistently annotated 7,620 GH sequences, out of 7,920 tested proteins (Table S1, Supplementary Data 1). For example, among the 327 biochemically characterized GH1s retrieved from the CAZy database, 325 (99.39%) of the sequences matched with PF00232 (i.e., “Glyco_hydro_1”) whereas the 2 mis-annotated GH1s were short fragments of sequences identified in cDNA libraries with biochemical characterization remaining elusive to date (e.g., myrosinase from *Sinapis alba*, CAA42536.1). Likewise, most GH families listed on the CAZy database were consistently identified using the GeneHunt approach (Table S1). Regarding GHs targeting cellulose, xylan, and chitin, the GeneHunt approach provided a systematic and consistent annotation for potential cellulases from GH5, GH6, GH7, GH8, GH9, GH12, GH44, GH48, for potential xylanases from GH10 and GH11, and for potential chitinases from GH18, GH19, and GH85. Conversely in a few GH families including GH16, GH22, GH52, and potential cellulases from GH45 and xylanases from GH30, fewer than 90% of the proteins were annotated consistently using the GeneHunt approach. Among the GH families that could not be identified were some families that have been reclassified (e.g., GH61, GH69) and some GH families with reduced number of sequences. Having no specific HMM profile, members of these families were eventually assigned to other GH families (e.g., GH 74, 82, 84, 86). Finally, some GH families were associated with several Pfam IDs corresponding to various subdomains (e.g., N- and C-terminal domains) such as GH30, GH36, GH49, and GH79. Globally, the sequences from these GH families with questionable Pfam-based annotation accounted for <3% of the analyzed sequences.

Next, we used the GeneHunt approach to identify GH domains in the M5nr database and investigated the exact domain associations among MDGHs (Table S1, Supplementary Data 2). The GeneHunt approach identified 322,068 unique protein sequences with GH domains among the 43,098,145 non-redundant sequences (~0.7%). The most abundant domains were from the GH13 α-amylase (n = 47,737), GH34 neuraminidase (n = 22,357), GH3 β-glucosidase (n = 21,503), and GH1 glucosidase (n = 17,715) families. Conversely, in some GH families we identified a reduced number of proteins including 16, 378, and 389 chitosanases from GH80, GH75, and GH46, respectively.

**Figure 1:**
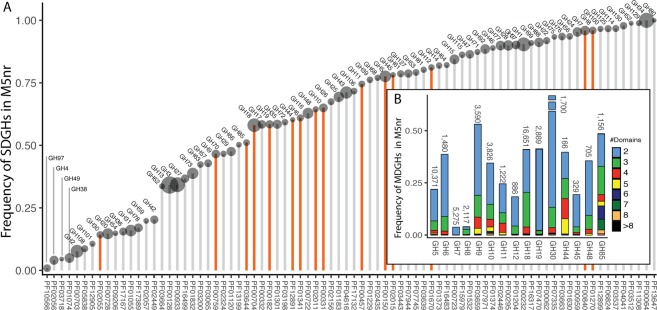
(**A**) Frequency of SDGHs identified in the M5nr database, size of dots mirrors the total number of identified domains (Supplementary Data). Highlighted in orange are the GH family detailed in (**B**). (**B**) Frequency of the complex MDGHs architecture in potential cellulases (i.e., GH5, 6, 7, 8, 9, 12, 44, 45, and 48), xylanases (i.e., GH10, 11, and 30), and chitinases (i.e., GH18, 19, and 85) identified in M5nr. Numbers correspond to the total number of identified domains in the M5nr database.

All the domains existed as single domain protein (i.e., SDGH). More precisely, >90% of the identified proteins with a domain from GH families 1, 7, and 34 were SDGHs (Fig. 1A), whereas most domains from GH families 2, 4, and 30, among others, were identified in MDGHs (Fig. 1A). Regarding the domains targeting cellulose, xylan, and chitin (Fig. 1B), most GH7s and GH8s were SDGHs, whereas ~25% of the domains from GH families 5, 11, 12, and 45 were found in MDGHs. Next, potential cellulases from GH families 6, 9, 44, and 48, xylanases from GH family 10, and chitinases from GH families 18, 19, and 85 were more frequently found associated with other domains. Finally, 86% of the identified GH30 domains were found in multi-domain proteins (mostly, in association with the subdomain GH30c, Fig. 1B).

**Figure 2:**
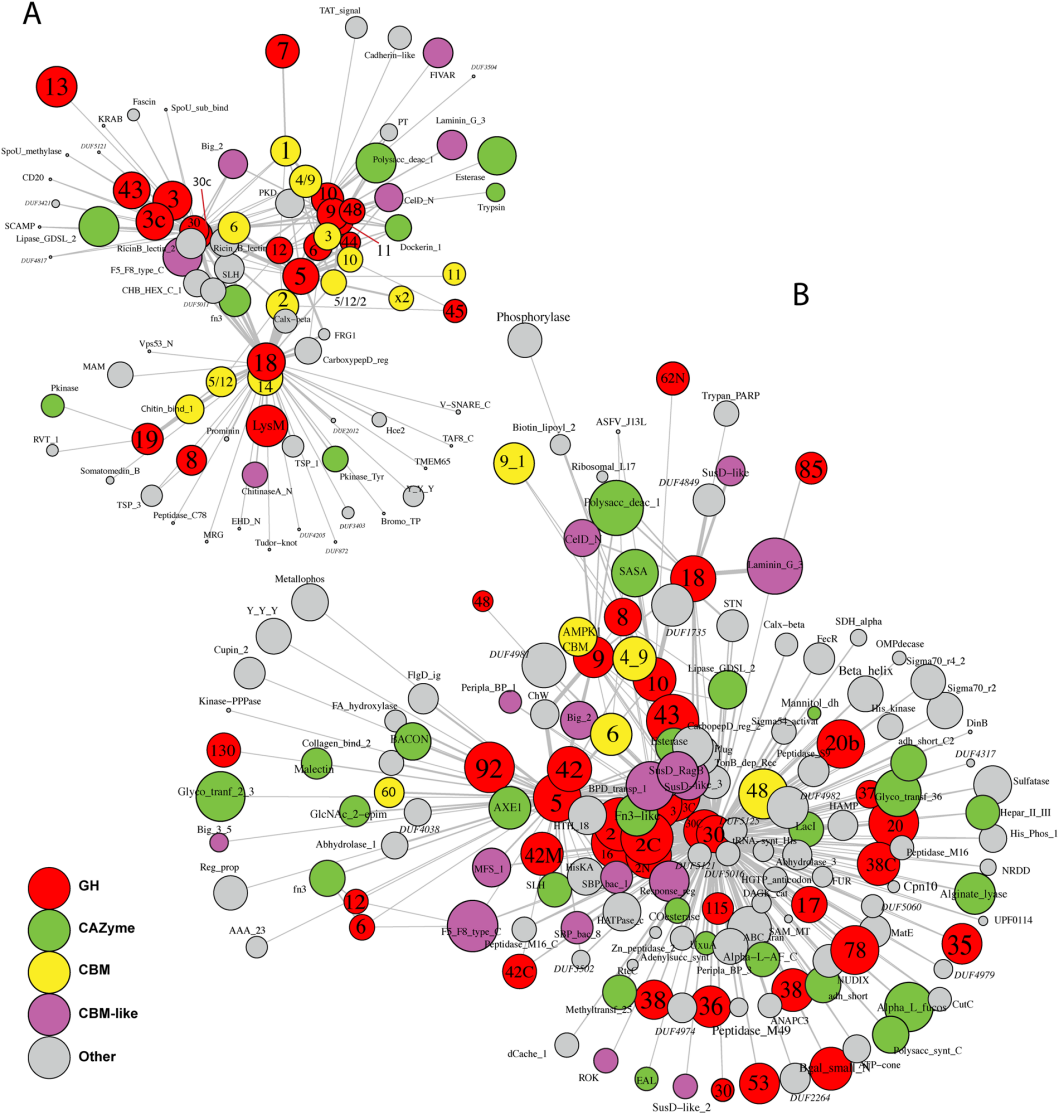
Association map for multi-domain cellulolytic, xylanolytic, and chitinolytic domains in the M5nr database (**A**) and in 129 combined assembled metagenomes (**B**), generated using igraph.

Associated with these GH domains, we found many potential non-GH CAZy domains including lipases (e.g., PF13472, PF00151) and polysaccharide deacetylases (e.g., PF01522) listed as carbohydrate esterase (CEs) in the CAZy database (Fig. 2A). We also identified many non-catalytic accessory CBMs and many other domains such as 6,672 F5/F8 type C_PF00754_ domains associated with GH20, GH2, GH5, GH13, GH43, and GH30 and 1,013 FIVAR_PF07554_ domains associated with GH85, GH20, GH31, GH43 and GH13 whereas 698 BIG_2_PF02368_ domains were associated with GH32, GH3, GH13, GH42, GH43, and GH10 among others.

**Figure 3:**
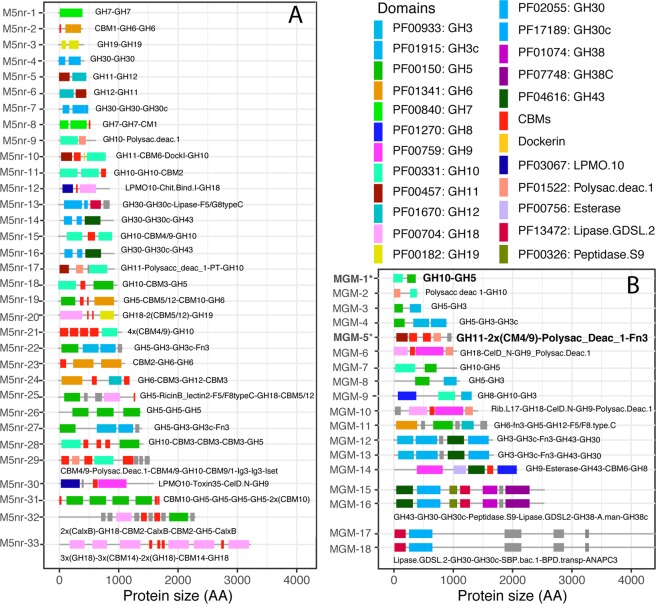
GeneHunt-based identification of new potential Multi-Activity GHs in the M5nr database (**A**) and in publicly accessible assembled metagenomes from MG-RAST (**B**). All identified proteins from MG-RAST (**B**) are from distinct metagenomes (Supplementary Data). CBMs (in red) include CBM1, 2, 3, 4/9, 5/12, 5/12-2, 6, 9-1, 10, 14, and chitin binding domain 1. MGM-1* and MGM-5* from assembled metagenomes were selected for further characterization (see text).

Potential MDGHs targeting cellulose, xylan, and chitin consisted mostly of only 2 associated domains. However, more complex proteins with >8 domains were also identified (Fig. 1B). Domains from GH9, GH10, GH44, and GH85 were the most frequently identified domains in these complex multi-domain proteins. For example, 82 different protein domains were identified associated with 3,619 GH9s. However, 27 domains were observed only once (e.g., Trypsin_PF00089_, LPMO_10_PF03067_, B-lectin_PF01453_) whereas 401 CBM3_PF00942_, 307 Dockerin-1_PF00404_, 187 CBM2_PF00553_, 1,305 CelD_N_PF02927_, and 89 fibronectin-3_PF00041_ were the most abundant domains associated with GH9, sometimes in complex associations (Fig. 3A).

In order to further investigate the diversity of MDGHs with potential for cellulose, xylan, and chitin deconstruction we next investigated the domain-co-occurrence network and focused on associations observed more than once (Fig. 2A). Regarding potential multi-domain cellulolytic enzymes, domains from GH5, GH6, GH9, GH12, GH44, and GH48 formed a large cluster also containing potential xylanases whereas GH8 was clustered with potential chitinases. In the large cellulolytic cluster, we identified 52 MDGHs with more than one GH5 (i.e., multi-GH5), and few containing up to 4 GH5 domains (Fig. 3A). Next, the GH5 domain was found in many multi-domain associations with non-catalytic domains (e.g., CBM2_PF00553_, CBM5/12/2_PF14600_) and several catalytic domains including GH6 (n = 6), GH9 (n = 3), GH10 (n = 4), GH11 (n = 11), GH12 (n = 16), GH18 (n = 12), and GH44 (n = 5) (Figs 2A and 3A). The other cellulases within this large cellulolytic/xylanolytic cluster displayed similar types of associations (Supplementary Data). Interestingly, we identified many MDGHs with domain repetition including 519 multi-GH9s, 8 multi-GH6s, 16 multi-GH7s, and 10 multi-GH44s, whereas no multi-GH45 nor multi-GH48 were identified. Beside these associations, only 3 out of 5,290 identified GH7 were associated with CBM1_PF00734_ whereas many GH45s (n = 339) were associated to CBM2_PF00553_ or CBM10_PF02013_. Finally, the 2,117 identified GH8, were found in none of the previously identified association, formed no multi-GH8, and clustered with potential chitinolytic domains (e.g., GH18).

Next, we identified two main clusters of multi-domain xylanases. The first one, with GH10 and GH11 clustered within the large cluster of previously identified potential cellulases. This cluster contained 30 multi-GH10s, 21 multi-GH11s, and 9 GH10-GH11 (Fig. 3A). We also identified several enzymes with non-catalytic accessory domains listed in the CAZy database such as CBMs (e.g., CBM1_PF00734_ and CBM4_9_PF02018_) and Dockerin_PF00404_ domains for cellulosome assembly (Fig. 3A). Several GH10s and GH11s were associated with other GH domains (e.g., GH5, GH12), or non-GH CAZyme domains such as Polysaccharide Deacetylase_PF01522_. Finally, several other domains, such as Esterase_PF00756_, were associated to GH10 and GH11 (Fig. 3A). The GH30, and its associated subdomain GH30c, formed a distinct and large cluster containing 56 multi-GH30s and displaying many domain associations not found with other xylanase domains. These included associations with other GH domains (e.g., GH3, GH13, GH43), non-GH CAZyme domains (e.g., CBM4/9_PF02018_, CBM6_PF03422_) and many other domains such Lipase GDSL-2_PF1347_, Ricin B lectin 2_PF1420_, and several domains with unknown function (e.g., DUF5011_PF16403,_ Figs 2A and 3A).

Regarding potential chitinases, we identified 277 multi-GH18s and 19 multi-GH19s. In addition, GH18 and GH19 were associated in one GH18-GH19 (Fig. 3A). Next, chitinases were associated with many of the previously listed GH domains (e.g., 12 GH18-GH5), and other GH domains such as LysM_PF01476_ (GH25, n = 4). In addition, several of these potential chitinases contained CBMs such as CBM14_PF01607_ (n = 254), CBM5/12_PF02839_ (n = 10), and CBM2_PF00553_ (n = 32). Finally, among the 1,163 listed GH85 domains, 7 multi-GH85s were identified. We also identified 221 Big-3_PF07523_, 227 F5_F8_type_C_PF00754_, and 176 FIVAR_PF07554_ associated to GH85, among others (Fig. 3A).

### Mapping of GHs in assembled metagenomes

Next, we used the GeneHunt approach to identify GH domains in 129 publicly accessible assembled metagenomes from MG-RAST (Table S1, Supplementary Data 3) and identified 200,257 GH domains corresponding to 160,790 proteins. Across datasets, potential α-amylases from GH13 were the most abundant domains (n = 28,135) followed by potential β-glucosidase from GH3 (n = 18,403), β-xylosidase/α-L-arabinofuranosidase from GH43 (n = 11,609) and β-galactosidase/β-mannosidase from GH2 (n = 10,571). As described for MDGHs in the M5nr database, we identified many domains associated to GHs including several well-known CBMs (e.g., CBM2), some non-GH CAZymes (e.g., glycosyl-transferase_PF00535_), and many catalytic (e.g., phosporylase_PF00343_, peptidase_M16_PF00675_) and non-catalytic domains (e.g.,DUF4979_PF16351_, Calx-beta_PF03160_) not listed on the CAZy database.

**Figure 4:**
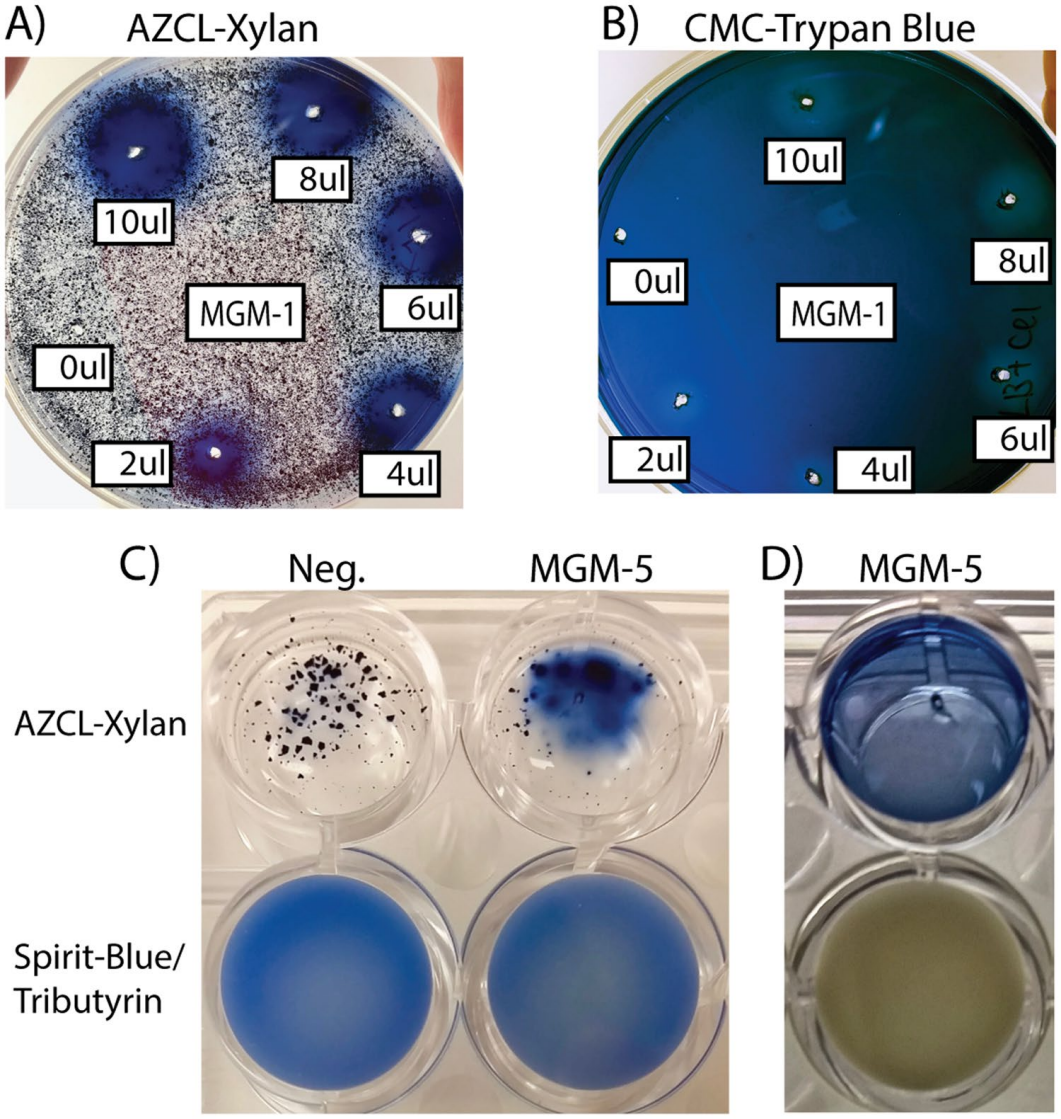
Biochemical activities of recombinant enzymes MGM1 (GH10-GH5) and MGM-5 (GH11-2 × CBM4/9-Polysacc.Deac.1-Fn3) (see Fig. 3). Before incubation, 0 to 10 µl of the MGM-1 extract in 20 mM Tris-HCl (pH 8.0) were mixed with 10 to 0 µl of 20 mM Tris-HCl (pH 8.0) to test the effect of protein concentration on AZCL-Xylan (**A**) and CMC-Trypan Blue (**B**). For MGM-5, 20 µl of cell extract in 20 mM Tris-HCl (pH 8.0) was incubated for 4 h (**C**) and 48 h (**D**) with AZCL-Xylan and Tributyrin. (Negative control was the cell extract of non-induced E. coli BL21(DE3):pet151B-MGM-5).

Next, potential domains for cellulose deconstruction were dominated by 5,004 GH5 domains distributed in 4,921 proteins and 82 multiGH5s. Next, we identified 1,459 GH9s, 85 GH6s (1 multiGH6), 9 GH7s, 746 GH8s (1 multiGH8), 71 GH12s, 132 GH44s, 17 GH45s (1 multiGH45), and 36 GH48s (4 multiGH48s). Noteworthy, beside potential cellulases with repeated domains, 97 MDGH cellulases displayed at least 2 potential catalytic domains targeting cellulose (e.g., GH9-GH8) (Figs 3B and 4B). Xylanase domains consisted in 2,175 GH10s (99 multiGH10s), 43 GH11s (1 multiGH11), and 1,547 GH30s (136 multiGH30s). Globally, 3,524 potential xylanases were identified; 3,288 proteins with only one xylanase domain, 2,224 being SDGH proteins, and 236 proteins with at least two potential domains for potential xylanase. Conversely, 1,300 potential xylanases were MDGHs.

Finally, chitinase domains including 3,286 GH18s (25 multiGH18s), 627 GH19s, and 247 GH85s (1 mGH85) corresponded to 3,888 proteins including 2,638 SDGHs and 1,250 MDGHs. Interestingly, 26 multi-domain chitinases displayed 2 potential chitinolytic domain.

Beside these multi-domain cellulases, xylanases, and chitinases we identified several potential cellulase-xylanase (e.g., GH5-GH11), cellulase-chitinase (e.g., GH5-GH18), xylanase-chitinase (e.g, GH11-GH18), and several other potential multi-activity enzymes (Fig. 3B, Table S3). Although many domain combinations were unique, some have been identified multiple times.

### Characterization of multi activity GHs

Overall, of the ~483,000 proteins sequences with at least one GH domains identified here, many contained non-catalytic accessory domain(s) and a few contained several catalytic domains. These corresponded to potential multi-activity proteins (i.e., MAGHs) with repeated domains such as proteins with multiple GH18 or multiple GH5 domains or proteins with distinct catalytic domains combined together (Fig. 3, Table S3). Some identified MAGHs were unique whereas some, even very complex, were identified multiple times and in different datasets. For example, a few very long and complex proteins with up to 7 distinct domains including 6 different potential catalytic domains were identified in several metagenomes (Fig. 3B). Although rare, being identified in multiple and unrelated datasets supports the biological origin of these complex proteins rather than *in silico* artifacts.

Based on the domain associations and knowing the function of several GH families, some identified MAGHs potentially displayed interesting synergies among the catalytic domains. Thus, in order to further demonstrate the biotechnological potential of these MAGHs we selected two unique proteins identified in distinct environmental datasets for sequence optimization and gene synthesis to proceed with heterologous protein production in *E. coli*.

First, MGM-1 (i.e., mgm4441594_JCVI_READ_1095454020156_1_1140_-) is a 377 amino acid protein, identified in a marine metagenome^36^, consisting of a potential cellulase from GH5 associated with a potential xylanase from GH10 (Fig. 3B). As expected, recombinant MGM-1 produced in *E. coli* was active on both AZCL-xylan and CMC (Fig. 4A,B).

Next, MGM-5 with 879 amino acids (i.e., mgm4491477_NODE_11875_length_6674_cov_6.617920_2824_5466_-), derived from a human gut sample^37^, contained a potential GH10-xylanase associated with a domain identified as a potential polysaccharide deacetylase and 2 CBM4/9s_PF02018_ (Fig. 3B, Table S3). As expected, recombinant MGM-5 hydrolyzed both AZCL-xylan and tributyrin (Fig. 4C,D).

## Discussion

The GeneHunt approach, using publicly accessible HMM-profiles from the Pfam A database^29^, can be used to identify the vast majority of characterized GHs (>98%) listed on the CAZy database^1^. The identification of rare or newly defined GH families such as the GH family 156 (introduced in October 2018)^1^ with just 7 identified sequences, having no HMM-profile available, is not yet possible using this approach^30^. For these families, until more sequences are identified and HMM-profiles created, similarity searches (e.g., BLAST^38^) using a custom database is an alternative (see Supplementary Data 1). However, as described for bacteria^2^ and fungal genomes^10^, the GeneHunt approach can identify the detailed architecture of most GH families in large database and in metagenomes. Instead of using a single-step HMMscan^28^ with a custom database (e.g., dbCAN^39^), the GeneHunt approach performs two sequential HMM-profile identifications. The first search, for all the protein sequences, is performed using a small custom HMM-profile database whereas the second scan, only for the potential positive hits derived from the first step, uses the entire Pfam A database^29^. This approach identifies all the domains associated with GHs including the ones not listed in the custom database while minimizing the number of sequence analyses, and thus is faster than a direct scan against the complete Pfam A database. This approach can be adjusted at will by searching new HMM-profiles in the small custom database, including HMM-profiles derived from dbCAN and other domains of interest such a susD-transporters_PF12741_ or lipases_PF00151_. In this context, the GeneHunt approach allows the identification and investigation of GH architecture in large databases such as sequenced bacterial genomes from the PATRIC database^2,40^, fungal genome from the Mycocosm database^10,41^ and MG-RAST^32^, as described here.

In addition to identifying 483,000 sequences for GHs in a curated database (CAZy database^1^), in a large non-redundant database (M5nr), and in 129 environmental datasets from MG-RAST^32^, we identified hundreds of accessory non-catalytic and catalytic domains associated with GH domains. Most identified MDGHs consisted of a GH domain associated with some non-catalytic accessory domain (e.g., CBM). Beside many well-characterized domains for non-GH CAZymes and carbohydrate binding modules (CBM)^1^, hundreds of domains are associated with GHs. Among others, the FIVAR_PF07554_ domain, found in various architectures, binds fibronectin and is sometimes linked to methicillin resistance^42,43^, whereas the F5/F8 type C_PF00754_ binds phospholipid on the surface of endothelial cells and adheres to glycoprotein in bovine milk^44,45^. The BIG_2 domain has been shown to be involved in cell-adhesion^46^. The systematic association of these various domains with GH domains provides insights on the modular nature of GHs in microbes. It has been shown that GHs can have multiple non-catalytic domains such as SusD-like domains_PF12741_^47^ or CelD_N_PF02927_ domains^48^, which mediate xyloglucan-binding for cellular intake or cellulose binding by certain cellulases respectively. These non-catalytic domains play a major role in substrate binding, and thus potentially, affect the overall catalytic efficiency of associated catalytic domains. The frequent association of poorly characterized domains (e.g. domains of unknown function - DUF) could be used to infer and test potential domain activities. Based on their frequency one could identify poorly characterized domains (e.g., DUF4979) systematically associated with specific GH domains to infer and test their function.

Additionally, a few MAGHs, displayed several catalytic domains and thus potentially combine distinct enzymatic activities. The vast majority of characterized GHs, including the ones listed on the CAZy database, targeting structural polysaccharides are single domain microbial GHs displaying low to moderate activity on natural substrates^30^. This highlights the need for multiple catalysts acting synergistically to support the efficient biomass deconstruction^20,49^. Linked multi-activity complexes such as cellulosomes^50^ and MAGHs^33,35,51^ display increased synergistic interaction amongst domains and represent an interesting alternative to complex mixtures of enzymes. Indeed, in cellulosomes and MAGHs, beside the additive effect of the catalytic domains there exist a proximity effect that reduces the diffusion of the catalytic domains relative to each other. However, although limited in the number of associated domains, MAGHs have the advantage of being stable covalent complexes, unlike cellulosome^52^ and the few characterized MAGHs display high hydrolytic activity^21,33,35,51^.

Identifying the complete set of domain combinations in MDGHs and MAGHs is a prerequisite to investigate the evolution of the protein domains from simple to complex multi-domain enzyme^53^. In addition, because the functions of many GHs families are conserved^1,30^, it is possible to infer how the combined domains could interact. Different types of synergy can be envisioned in MAGHs including linear pathway synergy (LPS), parallel pathway synergy (PPS), and debranching synergy (DS). In LPS the first catalytic domain is expected to release the substrate of the second catalytic domain whereas in PPS (e.g., MGM-1) the catalytic domains target distinct yet physically associated substrates. Finally, in DS the first catalytic domain cleaves the side groups in substituted polysaccharide thus increasing the accessibility of the polysaccharide backbone (e.g., MGM-5). Although rare, these MAGHs with multiple catalytic domains represent potential robust hydrolytic systems with reduced inhibition by the product^50,54^ and display proximity effect analogous to carbohydrate binding modules^3^. In addition, MDGHs with DS and including some esterase activity, could possibly disrupt the xylan-lignin complex and thus improve the xylan deconstruction by associated xylanases^55,56^. Finally, depending on the processivity (the enzyme’s ability to catalyze several consecutive reactions without releasing the substrate) of individual catalytic domain, some MAGHs could display unique modes of action^21,35^.

Finally, MAGHs combining distinct catalytic domains, while being encoded by single genes, can easily be edited (e.g., tagging the protein), cloned, and expressed in various hosts. In this context, the ever-growing number of accessible sequences-datasets (i.e., genomes and metagenomes) provides an unprecedented opportunity to identify new biotechnologically interesting catalysts. In addition, investigating the domain association in nature also highlights new ways to associate protein domains in order to take advantage of nature diversity for the purpose of synthetic biology.

## Methods

### GeneHunt approach

Briefly, the GeneHunt approach provides a Pfam-based domain-specific annotation of protein sequences^2^. More precisely, GeneHunt uses sequential HMM-profile searches^28^ to rapidly identify the detailed multi-domain organization of proteins containing a domain of interest (e.g., PF00150 for GH5). First, selected HMM-profiles for domains of interest (Table S1) derived from the Pfam-A database^29^ are searched (HMMsearch) in protein datasets. Then, the protein sequences of the potential positive hits are scanned against the entire Pfam-A database (HMMscan). The first search is fast and inaccurate, whereas the second scan identifies all the domains, not just the domains of interest, and removes the false positive hits. Although relatively slow, this second scan is performed on narrowed sets of sequences, thus making the overall process faster than a direct comparison of the entire dataset using the entire Pfam-A database. GeneHunt is publicly accessible on https://github.com/renober/GeneHunt_V1.

### Datasets

To test the GeneHunt approach, we first manually retrieved and reannotated 7,920 sequences for biochemically characterized GHs listed on the CAZy database^1^, as of June 2018 (Supplementary Data). Next, we retrieved and reannotated the M5nr database (ftp.metagenomics.anl.gov/, version 2013.12.15) containing ~43 × 10^6^ mostly microbial non-redundant protein sequences^31^. Finally, we retrieved the protein sequences from 129 publicly accessible assembled metagenomes from MG-RAST (Table S2) using the MG-RAST’s “application programming interface” (API)^57^.

### DNA synthesis and protein expression

The DNA sequences of two potential multi-activity GHs were first optimized for expression in *E. coli*, and cloned in the pET151 in order to incorporate the pelB signal peptide in the N-terminal end and a His-tag at the C-terminal end of the proteins (Thermo Fisher, Vista, CA., USA). The plasmids were then introduced into competent *E. coli* BL21(DE3) (Novagen, Madison, WI, USA). Heterologous protein expression was carried out in Lysogenic Broth at 37 °C for four hours by adding 0.4 mM isopropyl-D-1-thiogalactopyranoside (isopropyl-beta-thio-galactoside, IPTG) when the OD_600nm_ reached ~0.5. After centrifugation, the cell pellet was resuspended in 20 mM Tris-HCl (pH 8.0) and the cells were disrupted by sonication. Proteins from the cytoplasmic fraction were recovered by centrifugation at 20,000 × g for 40 min. Then enzymatic activities were tested qualitatively using chromogenic substrates. More precisely, azurin-cross linked xylan (AZCL-Xylan, Megazyme, Chicago, IL., USA) was used to detect xylanase, Trypan-Blue:CarboxyMethyl-Cellulose (CMC, Sigma-Aldrich, St Louis, MO., USA)^23^ was used for cellulase, and esterase activity was tested by incubating the extract with tributyrin in presence of the pH-indicator Spirit Blue^58^. Xylanolytic and cellulolytic activities were visualized after incubation for 4 hours at room temperature whereas tributyrin hydrolysis required a 48 hours incubation. Thus, in order to discriminate the recombinant activity from “residual activity” from the *E. coli* BL21(DE3) used for protein production, the cytoplasmic fraction of non-induced cells was used as negative control for tributyrin assay.

### Data processing and availability

Data were processed using R (Version 1.1.456) and the packages ggplot2, gplots, plyr, dplyr, reshape, reshape2, and igraph. PFam-based annotation of biochemically characterized GH listed on the CAZy database are in Supplementary File 1 and include the sequence ID, the taxonomic origin, the original annotation from CAZy, the EC-classification, and the GH domains identified using GeneHunt. Detailed GH-sequences annotation derived from M5nr database is in Supplementary File 2. Sequence from the M5nr database can be retrieved directly from the MG-RAST portal using MG-RAST API^57^ synchronous GET requests: http://api.metagenomics.anl.gov/m5nr/md5/SequenceID?sequence=TRUE).

Detailed GH-sequences annotation derived from M5nr database is in Supplementary Data 3. Sequences can be retrieved using MG-RAST API^57^ synchronous GET requests: http://api.metagenomics.anl.gov/download/mgmid?stage=650.

## Supplementary information

Supplementary Dataset 1

Supplementary Dataset 2

Supplementary Dataset 3

## Data Availability

All data generated or analyzed during this study are included in this published article (and its Supplementary Information Files). In addition, GeneHunt_V1.sh is publicly available on GitHub (https://github.com/renober/GeneHunt_V1).
